# Usability of Telehealth Technologies

**DOI:** 10.1155/2013/834514

**Published:** 2013-04-15

**Authors:** Zia Agha, Charlene R. Weir, Yunan Chen

**Affiliations:** ^1^Department of Medicine, University of California at San Diego, La Jolla, CA, USA; ^2^Department of Biomedical Informatics, School of Medicine, University of Utah, Salt Lake City, UT 84112-5775, USA; ^3^VA SLC GRECC, Salt Lake City, UT 84148, USA; ^4^Department of Informatics, Institute for Clinical and Translational Sciences, University of California, Irvine, CA, USA

In recent years, telehealth technologies are diffusing rapidly into all aspects of healthcare practices, ranging from traditional medical consultations to various patient-centric areas. These new uses bring a promise of integrating computerized support and Internet functionality into the deeper recesses of our community, addressing the intractable problems of access and population-specific health disparities. To ensure the promised benefits of these new telehealth systems, usability has become an ever-present and pressing issue for the research community.

Effective design and implementation of Health Information Technology (HIT) is foundational to providing health providers and patients effective, efficient, safe, and timely access to healthcare. The US Agency for Healthcare Research and Quality and the National Institute of Standards and Technology (NIST) have both stressed the need to measure and improve HIT usability. Usability, as defined by NIST, refers to “effectiveness, efficiency and satisfaction with which intended users can achieve their tasks in the intended context of product use.” This special issue presents original research articles that address the “usability” of telehealth systems. The articles presented illustrate how concepts of usability, cognitive support, and safety gain new complexities when HIT becomes more embedded into the everyday lives of different populations and settings. A range of populations can be served by telehealth, for example, those living in underserved urban communities, patients with a specific chronic illness, and older adults who are isolated from the community. Effective implementation of HIT can result in a dramatic increase in the reach of the healthcare system. Maximizing the benefit of telehealth applications requires deepening our understanding of usability in context, as each of the following studies illustrate.

The paper by S. George et al. aims to study the acceptability of telemedicine systems among urban underserved. This study uses the Diffusion of Innovation framework to examine the perceived advantages and readiness of telemedicine systems in underserved populations. The findings suggest that the adoption of new technologies is not merely a technical issue, but is situated in the social, cultural, and historical context. The same systems may not be successful in all settings, since the users have preperceptions about the technologies. The authors note that it is often nontechnical factors such as preconceptions about the technology that are related to patient satisfaction and effective patient-provider communication in a telehealth setting. The urban underserved poor have complex social relationships and unique needs and backgrounds that directly impact the use of telehealth. These insights have profound implications for other mHealth and eHealth systems, suggesting telehealth systems should be tailored and promoted according to users' backgrounds and preferences to avoid misperceptions and adoption failures. 

In contrast to the needs of a whole social group, the study by C. Stepnowsky et al. focuses on telehealth applications tailored to a specific disease. This study reports on an interactive website designed for patients with obstructive sleep apnea (OSA) in order to improve the adherence of continuous positive airway pressure (CPAP). It was found that if the design is tailored to the needs of individual users, the interactive portals could successfully improve adherence and patient education as well as foster clinical information. This study has valuable implications for the design of interactive systems designed for health management in chronic care illness.

In addition to the need to tailor systems to specific individuals or social groups, the paper by C. Diana et al. illustrates the need to address the diversity of settings in which mobile devices are used. This paper reports on a usability study of an application that collects symptom data for Fibromyalgia via a mobile device. The final goal of the project was to collect context-sensitive data on chronic pain using Experience-Sampling Methodology (ESM). Users in the study had low to medium experience with computers and touchscreens. To adjust to the complexities of using a system in all settings and times, it was suggested that the small mobile screen environment required stronger color contrast, redundant instructions, more response feedback, and more flexibility in positions for use. Although the study itself measured only qualitative information from a small number of users and a narrow set of concerns, the results do have some generalizability to the design of mobile applications. Future work on mobile devices might explore the ability to build in cross-senses instructions (e.g., audio and visual) and the importance of conducting usability studies in actual environments.

The paper by S. Spinsante et al. reports on the development of a systematic method of comparing types of health monitoring platforms targeted at older adults with heart failure. The authors compared 3 specific platforms in order to provide a generalizable method that could identify functional requirements for these types of information and communication technologies. The authors identify technical and user criteria for remote monitoring of physiological criteria, but information about how the model was actually used by older adults was not provided, limiting its generalizability. Nevertheless, the modeling of requirements and rating methods will be useful to generalize to other systems. Future work should focus on generalizability and integrating this system into other clinical areas as well as usability standards already in use.

The paper by M. Tabbara et al. reviews a new and growing area of wearable mHealth technologies. The use of wearable mHealth devices is a rapidly growing area of telehealth. In the consumer electronic space, there has been rapid growth in the number of personal wearable devices such as wearable activity, mood, and sleep monitors. The authors present their experience with a wearable device, the Swiss Limmex emergency wristwatch, designed as a medical emergency communication tool for older adults. Data on user's perceived needs, concerns, and willingness to adopt a simple device like the Limmex watch is presented. Because of the limitations of evaluating technologies in full context, little data on effectiveness, efficiency, or safety in an emergency scenario was provided. This study highlights the complexities of conducting usability research on wearable mHealth devices. 

By compiling this special issue, we hope to draw our readers' attention to the complexities and importance of addressing human factors and usability aspects of telehealth. Traditional telehealth research often includes supervised clinician-patient interactions that occur in the exam room. In this model, patients are passive recipients of telehealth care, and usability is often not addressed or is considered only from the clinicians' perspective. However, recent growth of patient-centric technologies, including mHealth and eHealth technologies (e.g., web portals, mobile phone applications, and wearable mHealth devices for patients), requires a reemphasis on usability from the perspective of patients and family caregivers. The special issue highlights studies that address issues of diversity, social interdependencies, individual difference and mobility, and the need for greater emphasis on usability as telehealth moves into this new era of diverse users. 

*Zia Agha* *Zia Agha* *Charlene R. Weir* *Charlene R. Weir* *Yunan Chen* *Yunan Chen*

